# Analysis of instantaneous brain interactions contribution to a motor imagery classification task

**DOI:** 10.3389/fncom.2022.990892

**Published:** 2022-12-15

**Authors:** Jorge Humberto Cristancho Cuervo, Jaime F. Delgado Saa, Lácides Antonio Ripoll Solano

**Affiliations:** ^1^Biomedical Signal Processing and Artificial Intelligence, Department of Electrical and Electronics Engineering, Universidad del Norte, Barranquilla, Colombia; ^2^SciFork SARL, Geneva, Switzerland; ^3^Grupo de Investigación en Telecomunicaciones y Señales, Department of Electrical and Electronics Engineering, Universidad del Norte, Barranquilla, Colombia

**Keywords:** brain interactions, correlation, Jaccard distance, classifier, static model, dynamic model, sliding window

## Abstract

The purpose of this study is to analyze the contribution of the interactions between electrodes, measured either as correlation or as Jaccard distance, to the classification of two actions in a motor imagery paradigm, namely, left-hand movement and right-hand movement. The analysis is performed in two classifier models, namely, a static (linear discriminant analysis, LDA) model and a dynamic (hidden conditional random field, HCRF) model. The impact of using the sliding window technique (SWT) in the static and dynamic models is also analyzed. The study proved that their combination with temporal features provides significant information to improve the classification in a two-class motor imagery task for LDA (average accuracy: 0.7192 no additional features, 0.7617 by adding correlation, 0.7606 by adding Jaccard distance; *p* < 0.001) and HCRF (average accuracy: 0.7370 no additional features, 0.7764 by adding correlation, 0.7793 by adding Jaccard distance; *p* < 0.001). Also, we showed that adding interactions between electrodes improves significantly the performance of each classifier, regarding the nature of the interaction measure or the classifier itself.

## Introduction

A brain-computer interface (BCI) is a communication and control scheme that sends messages and commands to the external world by interpreting brain waveforms (Wolpaw et al., 2002; Abiri et al., 2019). A regular BCI system has a brain monitoring system, a signal preprocessing stage, a stage for extracting features of the preprocessed signal, and a classification stage where features are decoded into commands or messages. Some manners of BCI activation are evoked potentials, brain rhythms, and motor imagery, among others (Scherer et al., 2007; Yao et al., 2022).

Different studies have suggested that combining frequency-temporal features with blinding source separation techniques improves the performance of classifiers in motor imagery paradigms significantly. One of the most outstanding techniques is the common spatial patterns (CSPs) that extract mutual features from a mixture of two populations by maximizing the different proportions of the variances in each population (Koles et al., 1990; Wang et al., 2021). In this way, a linear transformation (spatial filtering) is performed, preserving the number of sources but missing any interactions between electrodes. To compensate for the lack of information, a series of improvements have been introduced to CSPs, such as the filter bank CSP (FBCSP). In this study, the collected brain signal is processed by a set of different frequency band filters, and each filtered signal is processed by a CSP (Ang et al., 2008, 2012; Ferrero et al., 2021). Therefore, the contribution of a series of frequency bands is preserved while CSP extracts mutual features by the band. CSP and FBCSP can be applied over raw or preprocessed data, such as filtered data extracted from wavelet packet decomposition (Luo et al., 2019, 2020; Voinas et al., 2022), recombined data from CSP (Jalilpour Monesi and Hajipour Sardouie, 2019), or frequency data extracted from CSP (Oikonomou et al., 2020).

However, since CSP and FBCSP come from a linear transformation, the resultant operation only shows the projection of each electrode to the new space, such as its contribution to the total variance of the chosen population concerning the joint population (Bezdek and Pal, 1995; Wang et al., 2021). Furthermore, some recent studies have proposed to add interactions between electrodes—as correlation—to improve the CSP projection rather than being an input feature to a classifier by themselves (Zhang et al., 2013; Gubert et al., 2020; Ghanbar et al., 2021). Hence, the statistical contribution of these interactions is implicit as a CSP improvement and not as a feature. Other features, such as power bands, wavelet coefficients, and auto-regressive models, do not ever use any interactions between electrodes or brain zones (Aggarwal and Chugh, 2019; Mohdiwale et al., 2021).

The purpose of this study is to analyze statistically the contribution of the interactions between electrodes, measured here as correlation or Jaccard distance, as an additional input feature to the classification of two actions in a motor imagery paradigm, namely, right-hand movement or left-hand movement. The analysis uses two classifier models, namely, a static model and a dynamic model. The static model consists of a linear discriminant analysis classifier (LDA). The dynamic model consists of a linear conditional random fields (CRF) model, where features only interact with hidden variables rather than interacting with labels, named hidden CRF (HCRF). Also, the impact of using the sliding window technique (SWT) in the static and dynamic models is analyzed in this study.

## Methods

### Experiment and dataset description

The used dataset comes from the BCI Competition IV, dataset 2b (Leeb et al., 2007; Tangermann et al., 2012). Nine naïve volunteer subjects participated in the experiment. Each one was right-handed and had normal or corrected-to-normal vision. Each subject sat in an armchair and watched a flat screen placed 1 m away at eye level. Five sessions were performed for each subject: the first two without feedback and the last three with feedback. Each session consists of several runs preceded by 5 min of electrooculography (EOG) estimation at the beginning of each session.

The first two sessions used the following paradigm: a cue-based screening paradigm consisting of two classes, namely, left-hand movement and right-hand movement. Each session consisted of six runs, and each run had ten randomized trials by class, summarizing 120 repetitions per session. Each trial started with a fixation cross and a warning tone by 1 s approximately, followed by an arrow indicating either the left side or the right side for 1.25 s. Subsequently, the subject imagined the hand movement for 4 s. Next, there was a randomized pause for about 1.5 s to avoid adaptation.

The later three sessions made use of a smiley face for feedback, with four runs and 20 randomized trials by class and run, for a total of 160 repetitions per session. Each trial started with a gray smiley and a warning tone for ~1 s, followed by a cue period of 3 s, where the smiley displaced to the left or right. In this time interval, depending on the hand movement, the smiley became red when the subject was wrong and green when the moved hand was correct. Moreover, the mouth of the smiley also changed to sad (corners of the mouth downward) or happy (corners of the mouth upward), with wrong or right movement, respectively. Next, there was a randomized pause between 1 and 2 s. We employed data only from feedback sessions.

Waveforms were recorded from three EEG bipolar electrodes, namely, C3, Cz, and C4, with a frequency sample of 250 Hz. Fz channel was used as EEG ground. Later, data were bandpass filtered between 0.5 and 100 Hz, followed by a 50 Hz notch filter. In addition, the EOG is available from three monopolar electrodes and a similar amplification configuration. Additional details of the experiment are available in Leeb et al. (2007).

### Software implemented

Once the dataset was acquired, preprocessing, feature extraction, and classification stages were programmed in MATLAB^®^ 2021. HCRF was implemented by the Hidden-state Conditional Random Field Library version 2.0b (HCRF Library, 2011).

### Data preprocessing

During the imagery task, relevant information was collected between 3 and 6 s after the beginning of a trial. Then, the waveform was sub-segmented by sliding windows, varying the window size and the slide size along the experiments.

A single sub-segment can provide up to three types of features, namely, alpha/mu (8–12 Hz) and beta (15–25 Hz) power bands (Singh et al., 2011a,b, 2013; Pfurtscheller and McFarland, 2012) by each electrode, and a similarity (or distance) measure between a pair of electrode signals (C3–Cz, C4–Cz, or C3–C4). Then, power band data got scaled to an order of magnitude 10. Experiments used alpha and beta power bands as main features and similarity (or distance) measures as additional features. All features were obtained by using the SWT.

### Sliding window technique

The sliding window technique is a set of instructions executed over a subset of *k* consecutive values of **X**, being **X** = *{x*_1_*, x*_2_*, …, x*_*N*−1_*, x*_*N*_*}*, a discrete time series arrangement for *N* equally spaced time samples whose initial point is *x*_*i*_: **X**_**i, k**_ = *{x*_*i*_*, x*_*i*+1_*, …, x*_*i*+*k*−1_*, x*_*i*+*k*_*}*. Once the set of instructions was performed, the position of the initial point displaces by a distance Δ*i* and the algorithm takes another *k* points to set the new subset **X**_**i+**__**Δ*i, k***_ = {_*x*_*i*_**+**Δ*i*_,_*x*_*i*_**+**Δ*i*+1_, …,_*x*_*i*_**+**Δ*i*+*k*−1_,_*x*_*i*_**+**Δ*i*+*k*_}. The instructions process the new subset. The routine continues until the value of **X** corresponding to the final point *x*_*N*_ is reached. The size of the subset *k* and the displacement of initial point Δ*i* could be predetermined (Bandettini et al., 1993) or dynamically adaptive (BenYahmed et al., 2015).

The sliding window technique is convenient for data to get simple representations (BenYahmed et al., 2015; Hota et al., 2017) or find dynamic patterns (Mokhtari et al., 2019; Vergara et al., 2019) in a time series set. In this way, we propose to apply the SWT to increase the number of features obtained by trial: alpha and beta power values and the similarity (or distance) measures.

### Linear discriminant analysis

A discriminant classifier is a function that allocates an input vector *x* to one of *K* classes $C_{k}$ (Bishop, 2013). If we assume that *f*_*k*_(**x**) is a multivariate Gaussian with a vector of mean μ_*k*_ and a covariance matrix Σ_*k*_, we get the following discriminant function δ_*k*_(**x**) (Hastie et al., 2009):


$$\begin{array}{l} \delta_{k}\left(\text{x}\right)=-\frac{1}{2}\log|\sum_{k}|-\frac{1}{2}{\left(\text{x}-\mu_{k}\right)}^{T}\sum_{k}^{-1}\left(\text{x}-\mu_{k}\right)+\log\pi_{k} \end{array}$$

If each class has its covariance matrix Σ_*k*_, the discriminant function is quadratic by making the decision boundary between a pair of classes *k* and *l* as δ_*k*_(**x**) = δ_*l*_(**x**). However, if we suppose a shared covariance matrix Σ for all classes, the discriminant δ_*k*_(**x**) becomes linear (Hastie et al., 2009; Bishop, 2013). Notice that LDA-based classifiers are generative since they mostly assume Gaussian distributions in the data (Martens et al., 2011) and that LDA is a static model since time is not a relevant parameter for the classifier.

### Hidden conditional random fields

The conditional random field (CRF) classifier is a member of the probabilistic graphical models (PGMs) family. CRF represents complex distributions through products of local factors on small subsets of variables (Sutton and McCallum, 2011). Unlike other PGM models, CRF does not take the dependencies among entries but models directly the conditional distribution between input vectors *x* and output labels **y** as *p*(**y|x** ).

To expand the CRF models, we could add a set of hidden variables **h** = {*h*_1_, *h*_2_, …, *h*_*m*_} not observed during the training stage, associated with a singular output label *y* (Quattoni et al., 2007). Restricting the model to have disjoint sets of hidden states related to a certain value *y* of output label *h*_*j*_∈$H_{y}$, a hidden conditional random field (HCRF) takes the following form:


$$\begin{array}{l} p\left(\text{y}|\text{x}\right)=\frac{1}{Z}\underset{h_{j},h_{j^{\prime }}\in H_{y}}{\sum }\overset{T}{\underset{t=1}{\prod }}\exp\left\{\overset{K}{\underset{k=1}{\sum }}\theta_{k}f_{k}\left(h_{j,t},h_{j^{\prime },t-1},{\text{x}}_{t}\right)\right\} \end{array}$$

The partition function is a normalization function along all possible values of hidden variables along *y*. As CRF, a hidden variable *h*_*j, t*_ depends only on its predecessor *h*_*j′, t*−1_ and the corresponding input variables.

### Pearson correlation

Pearson correlation, defined for two zero-mean and real-valued random variables *x, y*, is the coefficient between the cross-correlation of the random variables *E*[*xy*] and the product of the square root of their variances σ_*x*_σ_*y*_ (Benesty et al., 2008, 2009):


$$\begin{array}{l} \rho \left(x,y\right)=\frac{E\left[xy\right]}{\sigma_{x}\sigma_{y}} \end{array}$$

We remark that the purpose of the study is to analyze statistically the contribution of the interactions between electrodes. In this study, we use correlation values to quantify the interaction between electrodes as a similarity measure.

### Jaccard distance

On the contrary, the Jaccard distance comes from its counterpart, the Jaccard Index *J*. The latter is a similarity measure for two sets *A, B*, defined as the coefficient between the size of the intersection |*A*∩*B*| and the size of its union |*A*∪*B*| (Fletcher and Isla, 2018). It can extend as the ratio between the measure of the intersection μ(*A*∩*B*) and its union μ(*A*∪*B*), with an arbitrary measure μ. If we define μ as the dot product between two multivariate variables *A, B*: μ(*A*∩*B*) = *A*•*B*, and μ(*A*) = ‖*A*‖^2^, being ‖*A*‖ the Euclidean norm of *A*, and using the relationship between the intersection and the union of two sets, the Jaccard Index *J* becomes:


$$\begin{array}{l} J\left(A,B\right)=\frac{A•B}{||A|{|}^{2}+||B|{|}^{2}-A•B}=\frac{A•B}{||A-B|{|}^{2}+A•B} \end{array}$$

With this definition, Jaccard Index takes values between −1/3 (when *B* = –*A*) and 1 (for *B* = *A*). However, for obtaining a non-negative metric, the Jaccard distance *J*_*D*_ = *1 – J* is defined as follows (Cha, 2007):


$$\begin{array}{l} J_{D}\left(A,B\right)=\frac{||A-B|{|}^{2}}{||A|{|}^{2}+||B|{|}^{2}-A•B}=\frac{||A-B|{|}^{2}}{||A-B|{|}^{2}+A•B} \end{array}$$

With this definition, Jaccard distance takes values between 0 and 4/3, becoming a non-negative measure. In this study, we use Jaccard distance values to quantify the interaction between electrodes as a distance measure.

### Performance metrics

A recurrent measure of performance for classification is *accuracy*. It estimates the closeness between measured or predicted values and their actual values (Clifford, 1985). For multiple classes, it is defined as the rate between the trace of a confusion matrix *H* and the total number of samples *N*_*s*_ (Schlögl et al., 2005):


$$\begin{array}{l} p_{0}=\frac{trace\left(H\right)}{N_{s}} \end{array}$$

where trace (*H*) is the number of samples correctly classified. The accuracy varies from 0 to 1, where 1 denotes a perfect classification. The study used five rounds of 3-fold cross-validation, where training data tune LDA and HCRF model parameters by an intern 4-fold cross-validation.

### Statistical analysis

A one-way randomized blocks ANOVA tested the statistical significance of differences between data with and without additional features. If the ANOVA test rejects the null hypothesis of statistical equality of averages, a Tukey-Kramer test performs a *post-hoc* comparison. The average value of each classifier and type of data is compared against the overall average accuracy, rejecting the null hypothesis if the average by class is greater than the overall average.

Also, a linear multiple-way randomized blocks ANOVA tested the statistical significance of differences in the data. The window size and the slide size were the tested parameters, and subjects were taken as randomized blocks in the model. If the ANOVA test rejects the null hypothesis of statistical equality of averages, a Tukey-Kramer test performs a *post-hoc* comparison.

## Results

The results of this study refer to the average performance obtained by each classifier, measured with the accuracy metric. All measures were obtained from the testing dataset of each subject.

### Results with the LDA model

Table 1 shows the accuracies obtained by comparing data with and without correlation features with the LDA classifier model. The 3-s trial was used without modifying other parameters. According to the ANOVA test [*F*: 34.922; degree of freedom (d.f.): 1; *p* < 0.001], the null hypothesis of average equality must get rejected, so we performed the *post-hoc* test. Their *p*-values are illustrated in Table 1 showing that only data with additional features are statistically significant regarding the average accuracy.

**Table 1:** Results of accuracy in the LDA model, with the presence or absence of interactions between electrodes.

**Subject**	**Type of interaction between electrodes**			
	**Correlation added**		**Jaccard distance added**	
	**Yes**	**No**	**Yes**	**No**
1	0.757	0.714	0.743	0.704
2	0.569	0.527	0.565	0.526
3	0.623	0.580	0.604	0.565
4	0.975	0.933	0.976	0.937
5	0.798	0.756	0.821	0.782
6	0.758	0.715	0.764	0.725
7	0.712	0.669	0.707	0.668
8	0.902	0.859	0.898	0.859
9	0.762	0.720	0.767	0.728
Average	0.762	0.719	0.761	0.722
Standard deviation	0.125	0.125	0.130	0.130
*P*-value	<0.001	1	<0.001	1
Overall performance	0.74		0.741	

Table 1 also shows the accuracies obtained by comparing data with and without Jaccard distance features with the LDA classifier model. As before, a one-way randomized blocks ANOVA tested the statistical significance of differences between data with and without additional features. According to the ANOVA test (*F*: 26.613; d.f.: 1; *p* < 0.001), the null hypothesis of average equality must get rejected, so we performed the *post-hoc* test. This indicates again that only data with additional features are statistically significant regarding the average accuracy.

The following step is to implement the sliding window algorithm in the data with power alpha and beta bands and additional features to obtain features dynamically. Hence, we used three sliding window sizes (0.5, 1, and 2 s) against the whole 3-s window and three slide sizes (0.125, 0.25, and 0.5 s) to compare the performance of the LDA classifier.

Table 2 shows the performance of the LDA model by window size and slide size by implementing the sliding window algorithm with correlation or Jaccard distance as additional features. According to the ANOVA test (*F*_window_size_: 46.61; d.f.: 3; *p*_window_size_ < 0.001; *F*_slide_size_: 144.60; d.f.: 2; *p*_slide_size_ < 0.001), the null hypothesis of average equality must get rejected, so we performed the *post-hoc* test. Results of Table 2 show that window sizes 2 and 3 s have the most outstanding performances. Meanwhile, slides 0.25 and 0.5 have the most statistically relevant values.

**Table 2:** Results of accuracy in the HCRF model, with the presence or absence of interactions between electrodes.

**Subject**	**Type of interaction between electrodes**			
	**Correlation added**		**Jaccard distance added**	
	**Yes**	**No**	**Yes**	**No**
1	0.752	0.712	0.753	0.711
2	0.596	0.557	0.600	0.558
3	0.568	0.529	0.571	0.529
4	0.991	0.951	0.992	0.950
5	0.790	0.751	0.809	0.767
6	0.778	0.738	0.788	0.747
7	0.763	0.724	0.750	0.708
8	0.919	0.880	0.919	0.878
9	0.831	0.792	0.832	0.790
Average	0.776	0.737	0.779	0.738
Standard deviation	0.135	0.135	0.135	0.135
*P*-value	<0.001	1	<0.001	1
Overall performance	0.757		0.758	

Considering the Jaccard Distance as an additional parameter, a similar procedure to correlation values was performed. According to the ANOVA test (*F*_window_size_: 30.28; 3 d. f.; *p*_window_size_ < 0.001; *F*_slide_size_: 137.73; 2 d. f.; *p*_slide_size_ < 0.001), the null hypothesis of average equality must get rejected, so we implemented the *post-hoc* test. Results of Table 2 show that window sizes 2 and 3 s have the most significant performance. Meanwhile, slides 0.25 and 0.5 have the most statistically significant values. Meanwhile, although the classifier performance is the highest when the slide size is 0.125 s, the average result is slightly better than the average performance with whole data, as illustrated in Table 2.

### Results with the HCRF model

**Figure 1:**
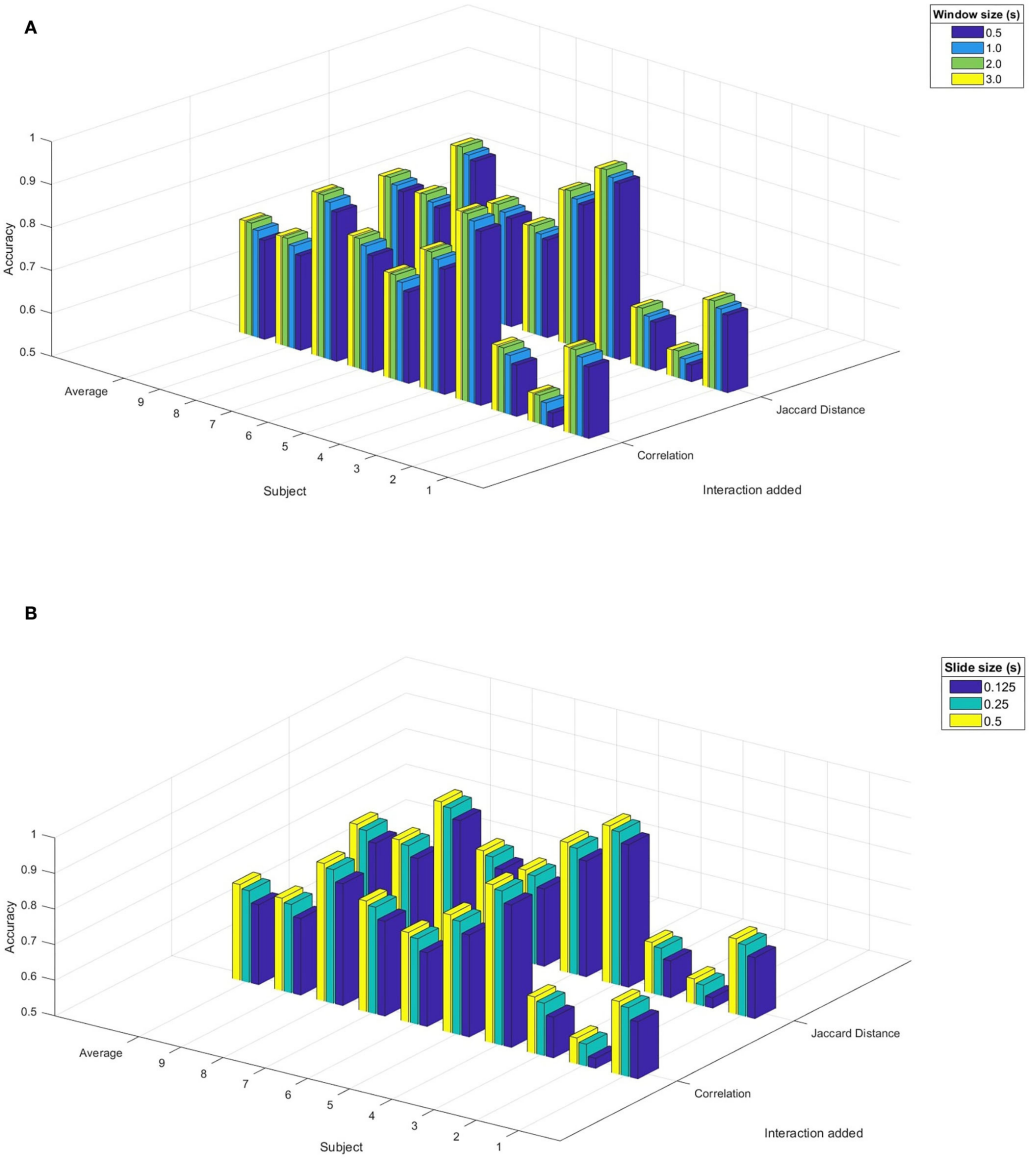
Results of accuracy in the LDA model with interactions between electrodes as an additional feature, by varying the window size or slide size in the sliding window algorithm. **(A)** Varying the window size. **(B)** Varying the size of the slide.

Since HCRF is a dynamic model regarding the LDA model, implementation of the sliding window algorithm is necessary to establish the corresponding timestamps of HCRF. To compare data with and without additional correlation features, we tested the model with two window sizes (0.5 and 2 s) and two slide sizes (0.125 and 0.5 s). Figure 1 shows the accuracies obtained by comparing data with and without correlation features implemented in the HCRF classifier. According to the ANOVA test (*F*: 117.232; d.f.: 1; *p* < 0.001), the null hypothesis of average equality must get rejected, so we performed the *post-hoc* test. Their *p*-values are illustrated in Figure 1, showing that only data with additional features are statistically significant regarding the average accuracy.

Figure 1 also shows the accuracies obtained by comparing data with and without Jaccard distance features with the HCRF classifier. As before, a one-way randomized blocks ANOVA tested the statistical significance of differences between data with and without additional features. According to the ANOVA test (*F*: 148.475; d.f.: 1; *p* < 0.001), the null hypothesis of average equality must get rejected, so we performed the *post-hoc* test. It indicates again that only data with additional features are statistically significant regarding the average accuracy.

The following step is to implement the sliding window algorithm in the data with power alpha and beta bands and additional features to get features dynamically. Hence, we used three sliding window sizes (0.5, 1, and 2 s) and three slide sizes (0.125, 0.25, and 0.5 s) to compare the performance of the HCRF classifier.

**Figure 2:**
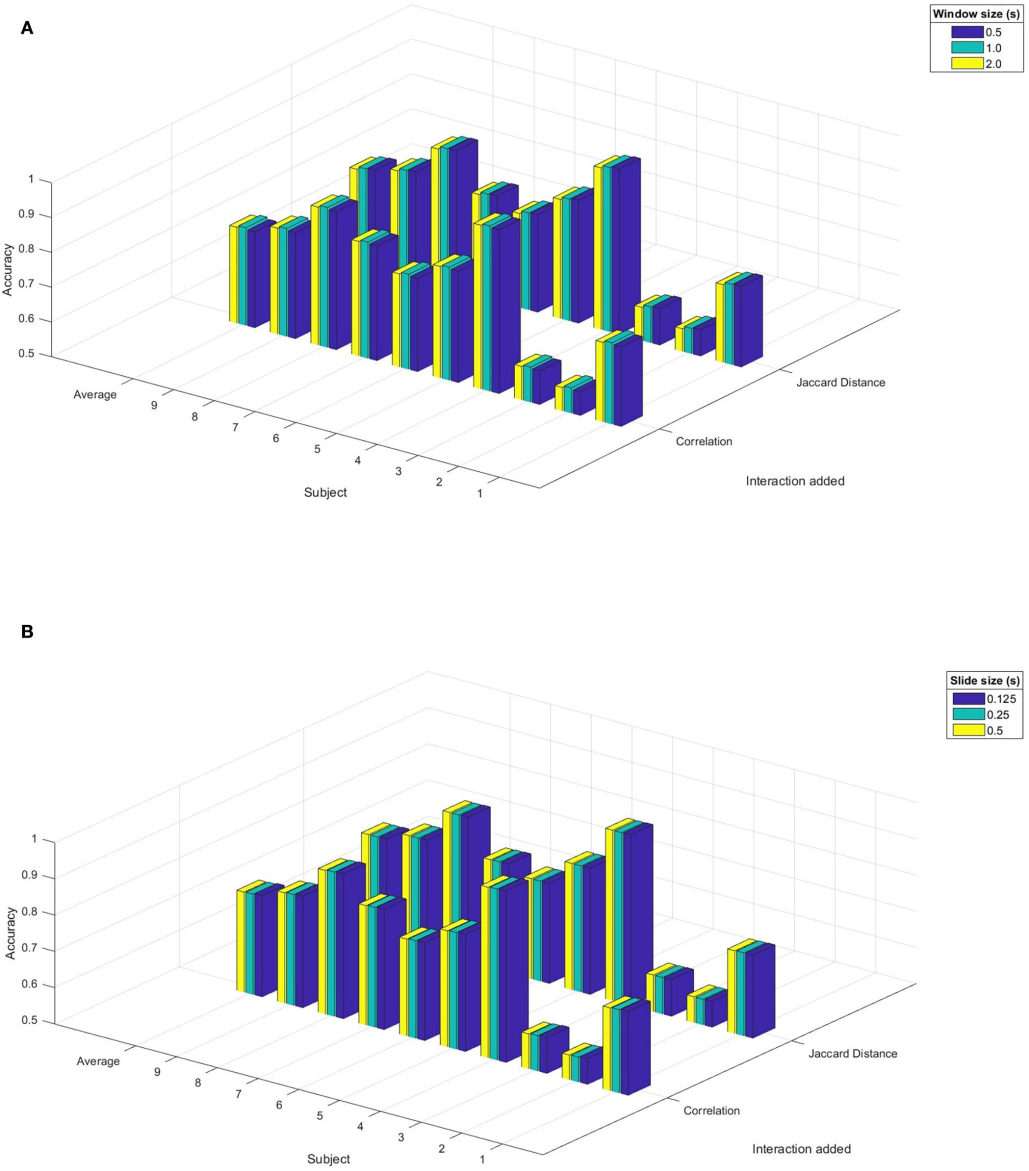
Results of accuracy in the HCRF model with interactions between electrodes as an additional feature, by varying the window size or slide size in the sliding window algorithm. **(A)** Varying the window size. **(B)** Varying the size of the slide.

Figure 2 shows the performance of the HCRF model by window size and slide size by implementing the sliding window algorithm with correlation as additional features. According to the ANOVA test (*F*_window_size_: 4.20; d.f.: 2; *p*_window_size_ = 0.016; *F*_slide_size_: 1.95; d.f.: 2; *p*_slide_size_ = 0.143), the null hypothesis of average equality must get rejected only for the window size, so we performed the *post-hoc* test. Results of Figure 2 show that a window size of 1 s has the most significant performance.

Considering the Jaccard distance as an additional parameter, a similar procedure to correlation values was performed. According to the ANOVA test (*F*_window_size_: 12.35; d.f.: 3; *p*_window_size_ < 0.001; *F*_slide_size_: 0.15; d.f.: 2; *p*_slide_size_ = 0.86), the null hypothesis of average equality must get rejected only for window size, so we implemented the corresponding *post-hoc* test. Results of Figure 2 show that the window sizes of 0.5 s have the most significant performance. Meanwhile, slides of 0.125 s have the most statistically significant performance, although it is not significant compared with the other slide sizes, as illustrated in Figure 2.

## Discussion

Results from Table 1 and Figure 1 suggest that adding correlation or Jaccard distance to the existing features improves significantly the performance of LDA and HCRF classifiers. It indicates that having available information on similarity or distance relations between channels gives additional knowledge about the classes that carry to a more accurate classification. However, it is the only behavior that the models have in common when electrode interactions get added to the features. Also, it is crucial to remark although correlation and Jaccard distance are quantifications of interactions with distinct attributes, we get a performance improvement for both measures. It means that adding interactions between electrodes significantly improves the performance of given classifiers, independent of the nature of the interaction measure.

In the LDA model, results from Table 2 show that only a few additional pieces of information provided by the SWT are enough to improve the classification. It is performed by adding brain interactions from 2 s sliding windows and displacements of 0.5 s, or even with the whole trial with no shifts. It is due to the nature of the LDA model, where a dimensionality reduction of data to 1 dimension is necessary to perform the discrimination analysis (Bishop, 2006). With more features added, the complexity of data gets enhanced because of their dimensionality. Since dimensionality reduction always implies loss and distortion of information, preserving most of them in a tractable processing core is mandatory (Gracia et al., 2014; Zenil et al., 2016). One mode is handling data with reduced dimensions before the dimensionality reduction step, which preserves information with less distortion and loss. It happens by using brain interactions from 2 s or higher sized windows and displacements of 0.5 s.

On the contrary, the HCRF model has different behavior. Results from Figure 2 show that the window size is the most relevant parameter to implement the SWT. Simultaneously, the change in the slide size had no or less effect on the classifying performance. It indicates that information from window size smaller than or equal to 1/3 trial size is more relevant than the one coming from longer windows. It also leads to the longest available window displacement usage without losing relevant information, reducing the dimensionality and, hence, the input size of the features to the model.

In summary, although electrode interactions do not contribute significantly to classification in a multiclass task by themselves (Miller et al., 2014), this study proved that their combination with temporal features provides significant information to improve the classification in a two-class task, such as motor imagery. Also, we showed that performance improvement is independent of the nature of the interaction measure. The future direction of the study point is to use the electrode interactions as additional features in motor imagery tasks with more than two classes, more than three electrodes, and dividing the frequency power bands into smaller sizes.

## Data availability statement

Publicly available datasets were analyzed in this study. This data can be found here: https://www.bbci.de/competition/iv/.

## Ethics statement

Ethical review and approval was not required for the study on human participants in accordance with the local legislation and institutional requirements. The patients/participants provided their written informed consent to participate in this study.

## Author contributions

JC: experiments conducting, writing of first draft, and statistical analysis performance. JD: design of experiment protocol, experiments, and state-of-the-art supervision. LR: statistical analysis, results supervision, and paper structure. All authors contributed to manuscript revision, read, and approved the submitted version.
